# Maximising adherence to study protocol within pharmaco-rehabilitation clinical trials

**DOI:** 10.1186/1745-6215-12-S1-A133

**Published:** 2011-12-13

**Authors:** Suzanne Hartley, Sharon Ruddock, Bipin Bhakta, John Pearn, Lorna Barnard, Alison Fergusson, Dean Langan, Amanda Farrin

**Affiliations:** 1 Clinical Trials Research Unit, University of Leeds, Leeds, LS2 9JT, UK; 2 Academic Department of Rehabilitation Medicine, University of Leeds, LS2 9JT, UK

## Background

The Dopamine Augmented Rehabilitation in Stroke (DARS) trial is a double-blind placebo controlled trial investigating impact of co-careldopa/placebo in combination with routine NHS occupational/physical therapy on functional outcome in people with acute stroke. The trial involves participants taking Investigational Medicinal Product (IMP)/placebo prior to therapy sessions while in the acute stroke unit and following hospital discharge

Stroke survivors may have significant residual impairments such as weakness, aphasia, visual disturbance, cognitive problems and mood disorders which may affect their ability to comply with DARS medication/therapy schedule.

## Objectives

To identify issues in adherence and retention to medication/therapy schedule and implement processes to maximise compliance across hospital and community settings.

## Methods

Key aspects of compliance with the medication/therapy schedule included (a) ensuring IMP packaging was clear and useable by people with hemiparesis; (b) IMP was taken at the correct time in relation to therapy intervention.

### Packaging & labelling

We involved 19 stroke survivors in small group discussions about different aspects of IMP labelling and packaging. Different examples of IMP packaging(developed using NPSA guidance) were presented and preferences/opinions were obtained through standardised questionnaires.

### Compliance with therapy / medication schedule

We developed a DVD for the participant/carers to view in their home environment to provide an audio visual aid to supplement the Patient Information Sheet. The DVD included voice over explaining trial processes in particular the therapy/IMP schedule, safety issues and contact details. IMP/therapy schedule compliance was also discussed with community therapists.

## Results

The patient feedback was incorporated into IMP packaging to allow one handed opening and prompts for adherence to IMP schedule.

The DVD content is presented in a manner accessible to patients with aphasia or hemisensory neglect and uses graphics to illustrate abstract concepts such as randomisation. The DVD will be given to trial participants as part of the recruitment information pack.

A process has been implemented for the therapist (a) to call the patient 45 minutes before the therapy session to remind the patient to take their IMP and (b) to conduct a compliance check at each therapy session.

## Conclusions

The trial opened to recruitment in May 2011. An evaluation of the above approaches has been build into the trial follow up outcome visits. Recruitment, adherence to trial protocol and patient satisfaction with information provided will be used as outcomes to judge impact of above strategies.

